# Supplementary figures and images for: The GGDEF-EAL protein CdgB from Azospirillum baldaniorum Sp245, is a dual function enzyme with potential polar localization
Source: PLoS One. 2022 Nov 23;17(11):e0278036. doi: 10.1371/journal.pone.0278036 (PMC9683572; doi:10.1371/journal.pone.0278036)

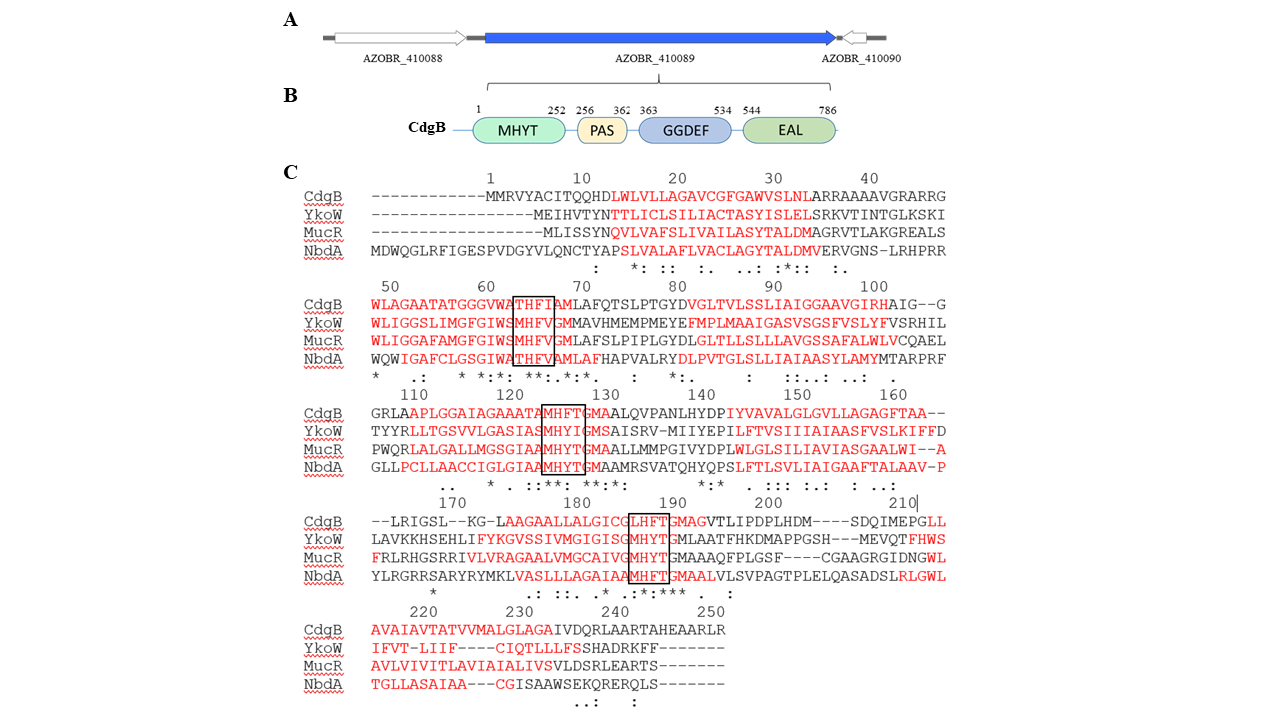

Supplement: S1 Fig — (A) Genetic context of the cdgB gene (AZOBR_410089) which codifies the CdgB protein is composed of a putative lysR gene upstream (AZOBR_410088) and a gene of unknown function downstream (AZOBR_410090). (B) Schematic representation of the domain structures of CdgB. The numbers represent the start and end amino acid of the predicted domains based on SMART database. (C) The Sequence alignment of the MHYT domain of CdgB with other c-di-GMP metabolizing proteins harboring an MHYT domain: the YkoW protein of B. subtilis, NbdA and MucR of P. aeruginosa. The three MHYT motifs are labeled by black squares and the transmembrane regions determined by the SMART and TOPCONS programs are highlighted in red. (TIF) [file pone.0278036.s001.tif]

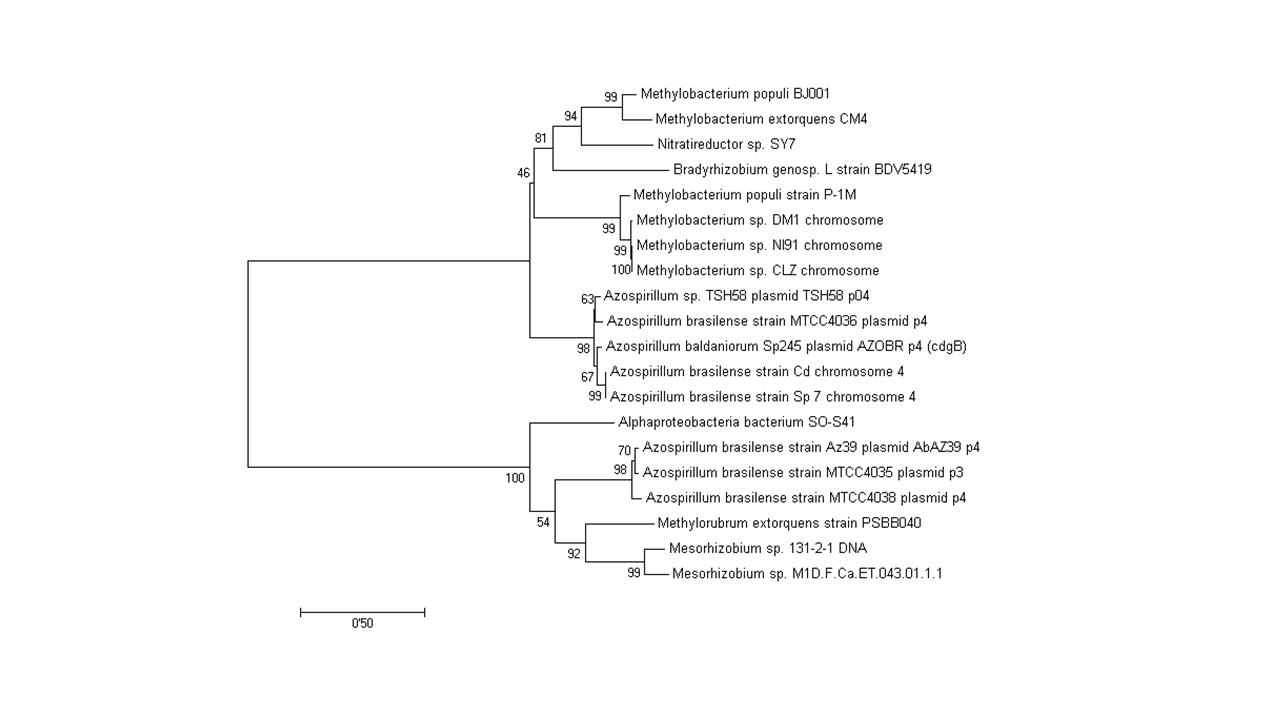

Supplement: S2 Fig — Phylogenetic tree showing the cdgB gene of A. baldaniorum Sp245 conserved in the alphaproteobacteria class. Nucleotide sequences of cdgB gene and homologous related genes were aligned by Clustal Omega. Phylogenetic tree files were generated by MEGA version VII. The phylogenetic tree was inferred using the Maximum Likelihood method and Tamura-Nei substitution model, with 1000 bootstrap replications. (TIF) [file pone.0278036.s002.tif]

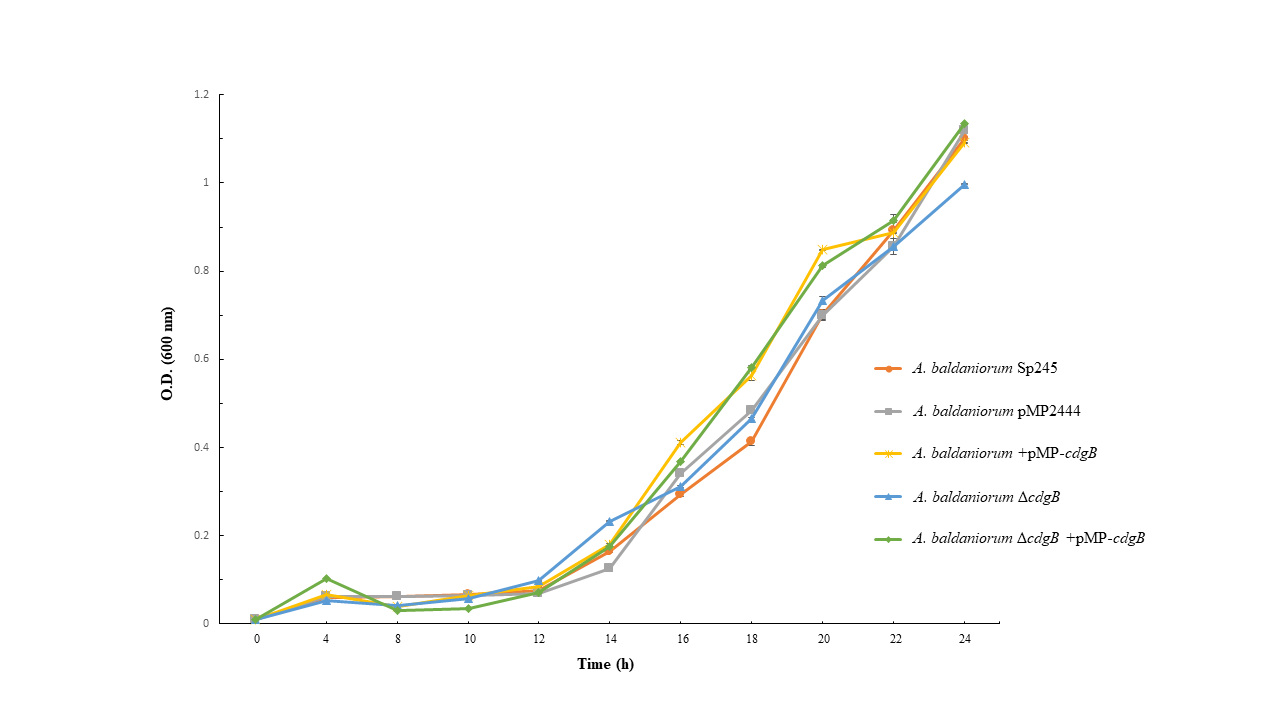

Supplement: S3 Fig — The strains were grown in NFB* media. Growth was measured at OD600 nm every 2–3 hours. Cultures were kept for 24 h at 30°C under agitation (150 rpm). Data showed is representative from three independent cultures of each strain. (TIF) [file pone.0278036.s003.tif]

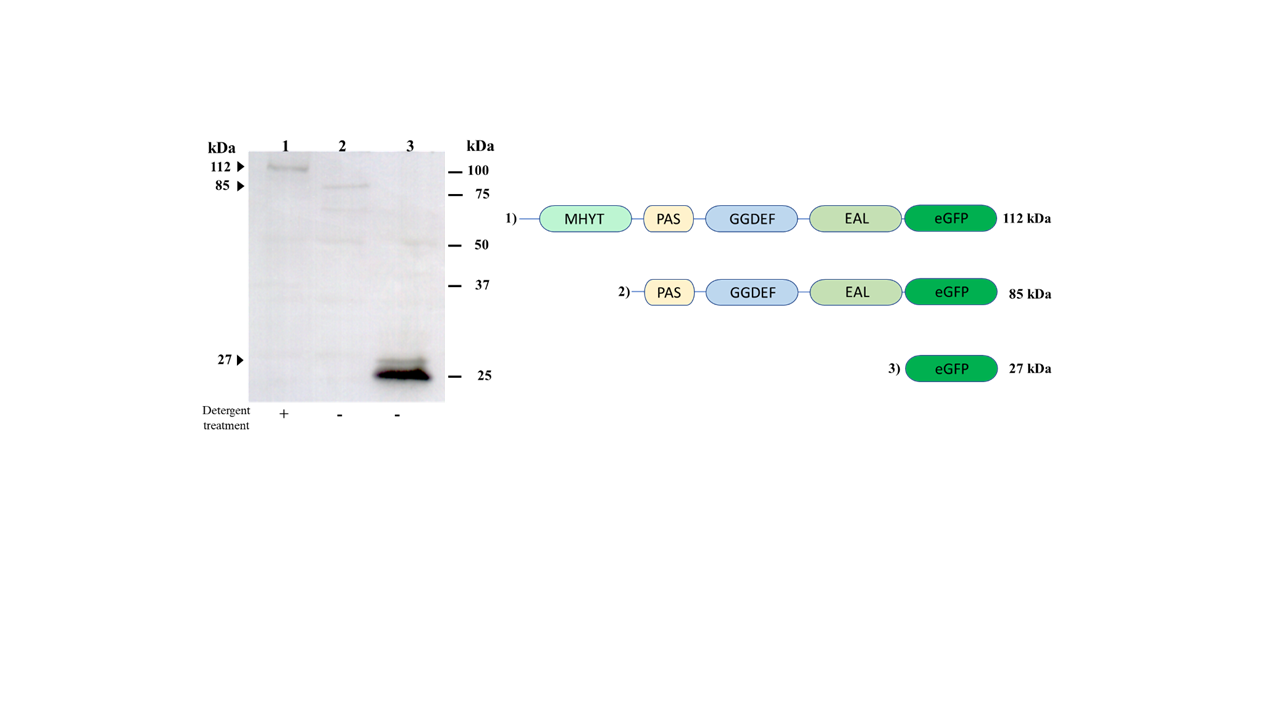

Supplement: S4 Fig — Detection by Western blotting of CdgB::eGFP fusion proteins from soluble and detergent treatment protein extracts of A. baldaniorum cdgB::egfp strain, and the truncated protein variant from the ΔMHYT-cdgB::egfp derivative strain, harboring the plasmid pMP+cdgBΔMHYT. The GFP D5.1 Rabbit mAb, HRP Conjugate against GFP was used. Lane 1 A.baldaniorum cdgB::egfp; lane 2 ΔMHYT-cdgB, variant protein; lane 3. A. baldaniorum pMP2444. The relative molecular weight (MMr) is indicated as kDa. The black arrows indicate MMr in kDa. The corresponding figures represent the proteins indicate in each well. (TIF) [file pone.0278036.s004.tif]

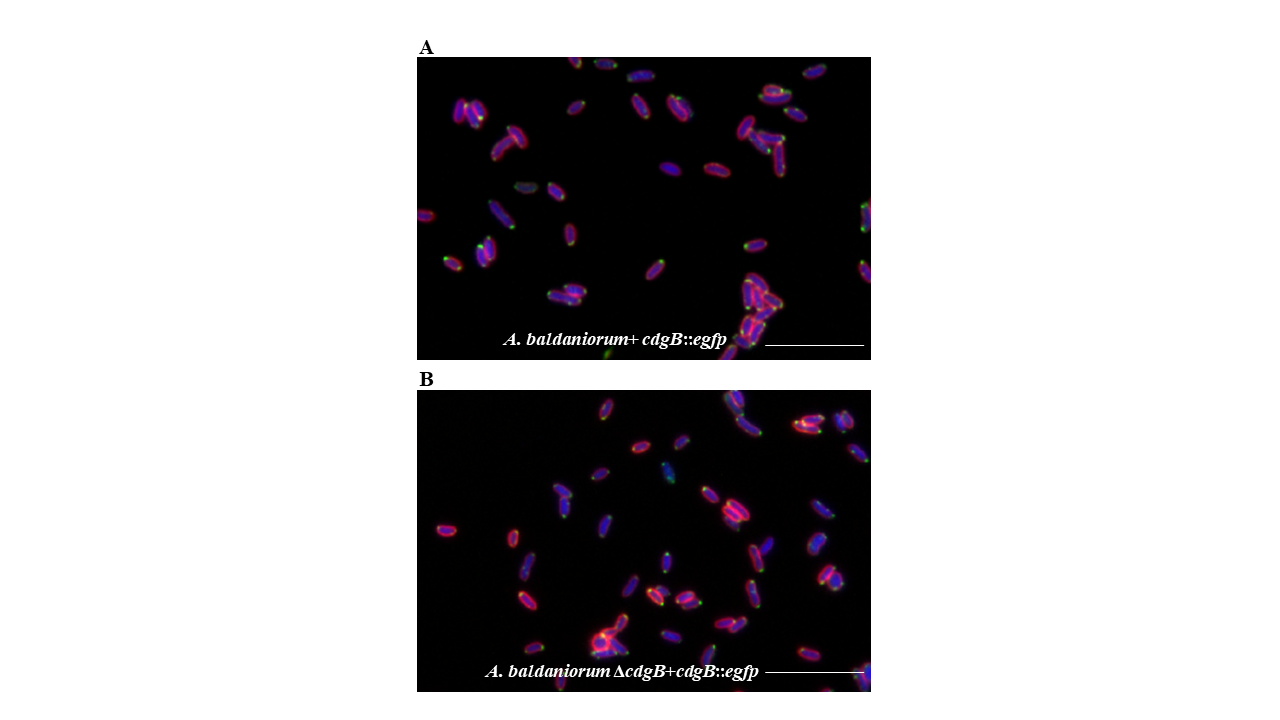

Supplement: S5 Fig — A) A. baldaniorum+ cdgB::egfp, B) A. baldaniorum ΔcdgB+cdgB::egfp were grown in NFB* medium. Photographs of the GFP fusion protein in WT and ΔcdgB strains were detected using a fluorescence microscope (TE 2000U; Nikon). Different subcellular locations of protein CdgB-eGFP including polar, bipolar, multisite were visualized. In red is showed membrane lipids with FM4-64FX, in green the CdgB-eGFP fusion, and blue bacterial nucleoid staining with DAPI. The images are representative of three biology repeats. Scale bar correspond to 10 μm. (TIF) [file pone.0278036.s005.tif]
