# Supplementary material for: The GGDEF-EAL protein CdgB from Azospirillum baldaniorum Sp245, is a dual function enzyme with potential polar localization
Source: PLoS One. 2022 Nov 23;17(11):e0278036. doi: 10.1371/journal.pone.0278036 (PMC9683572; doi:10.1371/journal.pone.0278036)
Supplement: Attachment — Submitted filename: Specific comments.docx [file pone.0278036.s007.docx]

**Specific comments:**

**Minor comments**

-- Line 26: change “stablishing” to establishing.

-- Line 27: in silico italicize

-- Line 30: Sp245. It inhibits…

-- Line 34: MHYT domain negatively affects extracellular…

-- Line 37: under certain conditions…

-- Line 45: plays a central role in microbial cellular behaviors in response to the environment…

-- Line 53: delete “,” after intra-

-- Line 59: Bioinformatic

-- Line 81: CdgB DGC and PDE activity under same conditions or different? Please mention.

-- Line 85: What is PGPR? First time used abbreviation should be elaborated.

-- Line 113: Gentamicin

-- Line 245-46: How did you measure the color intensity? Please mention

-- Line 311: proposed to be…

-- Line 449: riboswitches? Or riboswitch? Please specify one or multiple.

-- Line 508: *Bacillus subtilis*, although…

**Major comments**

-- Line 144: For overexpression of *cdgB* was cloned along with its native promoter? Or the ORF of the gene was cloned in frame under the p*lac*? If the native promoter was included, then there is a possibility of additional regulation at the *cdgB* promoter. The phenotypes observed in *A. baldaniorum* might include additional effects of *cdgB* transcriptional regulation. The experiments with overexpression of *cdgB* will benefit from constitutive expression under *lac* promoter.

**Additional suggestion:** to support the expression of all the native and mutated variants of CdgB, a western blot of tagged protein would be strong evidence for full expression of the protein variants. This would confirm that the phenotypes observed was not because of any artifacts.

-- Fig 4, 5 and 7: Biofilm, EPS production and swimming assay- the T test and the annotations on the graph for statistical significance is confusing and doesn’t compare all the samples tested. A One-way ANOVA followed by a multiple comparison test (Like Tukey’s test) would actually compare all the sample sets and give the readers a clear idea of the statistically significant differences among each of the samples. Also, was T-test performed between each of the samples or just between each control and their corresponding tests samples? Need a mention. The asterisks and the labels above the bars in the graphs are not clear which samples are being compared. This can be explained briefly in figure legends.

-- The c-di-GMP differences in various strains and overexpression of *cdgB* was measured mostly by qualitative methods. A quantitative measurement of cellular c-di-GMP would be helpful to understand the dominance of either DGC or PDE activity of CdgB under different conditions.

-- A quantification of cellular c-di-GMP level in *A. baldanorium* strains is required. The differences of phenotypes (like biofilm, swimming, etc.) observed in the strains because of deletion or overexpression of *cdgB* is caused by variation of c-di-GMP level, needs to be established.

-- The DGC activity of CdgB increased by overexpression in *E. coli* but the reverse in *A. baldanorium.* Is it host specific? Requires an elaboration in the discussion section. A quantitative measurement of cellular c-di-GMP can be done with overexpression of *cdgB* in both *A. baldanorium* and *E. coli* under the same or similar condition and a comparison can be shown to determine the concentration-dependent catalytic activity of CdgB. Then conclusion can be made whether the DGC activity of CdgB in *E. coli* is host-specific or conditional.

-- Deletion of *cdgB* resulted in a different phenotype compared to overexpression in *A. baldanorium*. This suggests that high cellular concentration of CdgB increases its PDE activity in *A. baldanorium*. This means that increased transcription and subsequently translation of *cdgB* is responsible for reduced c-di-GMP in the bacterium in response to the environment. An experiment showing transcription of *cdgB* and also its translation under different conditions such as presence and absence of NO, O_2_ and CO_2_ followed by measurement of intracellular c-di-GMP levels in *A. baldanorium*. It can be beneficial and complement the data shown in the manuscript. This experiment can be used to determine if the PDE and DGC activity of CdgB is dependent on its intracellular concentration or environmental signals, since the signals perceived by this protein is to be determined in future.
