# Supplementary material for: The GGDEF-EAL protein CdgB from Azospirillum baldaniorum Sp245, is a dual function enzyme with potential polar localization
Source: PLoS One. 2022 Nov 23;17(11):e0278036. doi: 10.1371/journal.pone.0278036 (PMC9683572; doi:10.1371/journal.pone.0278036)
Supplement: Attachment — Submitted filename: Response to Reviewers.docx [file pone.0278036.s008.docx]

**Dr. Prof. Ching-Hong Yang**

**Academic Editor**

**PLOS ONE**

Dear Prof. Yang, we are interested in published our work in PLOS ONE journal that is a research article, which is entitled “**The GGDEF-EAL protein CdgB from *Azospirillum baldaniorum* Sp245, is a dual function enzyme with potential polar localization”** by Víctor I. Viruega Góngora, Iris S. Acatitla Jácome, David Zamorano-Sánchez, Sandra R. Reyes Carmona, María L. Xiqui Vázquez, Beatriz E. Baca, and Alberto Ramírez-Mata*. We think that our study provides new insights into the contribution of genes associated to biosynthesis of the cyclic di-GMP in the plant growth promoting rhizobacteria *A. baldaniorum* Sp245*.* Here enclosed a summary of the study and revision requested: In this manuscript, we present in silico analysis and experimental characterization of the function of CdgB (AZOBR_p410089), a predicted MHYT-PAS-GGDEF-EAL multidomain protein from *A. baldaniorum* Sp245. When overproduced, CdgB behaves predominantly as a c-di-GMP phosphodiesterase (PDE) in *A. baldaniorum* Sp245, it inhibits biofilm formation and extracellular polymeric substances production and promotes swimming motility. However, a CdgB variant with a degenerate PDE domain behaves as diguanylate cyclase (DGC). This strongly suggest that CdgB is capable of dual activity. Variants with alterations in the DGC domain and the MHYT domain affect inhibition of extracellular polymeric substances production and induction of swimming motility. Surprisingly, we observed that overproduction of CdgB results in increased c-di-GMP accumulation in the heterologous host *Escherichia coli*, suggesting that in certain conditions, the WT CdgB variant can behave predominantly as a DGC. Furthermore, we also demonstrated that CdgB is anchored to the cell membrane and localizes potentially to the cell poles. This localization is dependent on the presence of the MHYT domain.

We appreciate too much the constructive criticism and suggestions made by reviewers. We hope that our manuscript will allow it to be considered for publication in the prestigious PLOS ONE journal.

Journal requirements:

We want to clarify our financial disclosure as following; Consejo Nacional de Ciencia y Tecnología (CONACyT), Grants INFRA-2014-01-225923, and the financial support of Vicerrectoría de investigación y estudios de posgrado (VIEP).

The funders had no role in study design, data collection and analysis, decision to publish, or preparation of the manuscript.

We want to declare that Víctor I. Viruega Góngora received a scholarship of CONACYT for his Ph.D. studies.

We added the blot/gel image data in supporting information, we upload a new copy of Figures 2, 3 and 7.

We look forward to receiving news from you soon.

Kind regards,

**Alberto Ramírez Mata PhD**

Corresponding author

Centro de Investigaciones en Ciencias Microbiológicas

Benemérita Universidad Autónoma de Puebla

e-mail alberto.ramirez@correo.buap.mx

**Specific comments:**

**Minor comments**

-- Line 26: change “stablishing” to establishing. Corrected

-- Line 27: in silico italicize. Corrected

-- Line 30: Sp245. It inhibits… Corrected

-- Line 34: MHYT domain negatively affects extracellular… Corrected

-- Line 37: under certain conditions… Corrected

-- Line 45: plays a central role in microbial cellular behaviors in response to the environment… Corrected

-- Line 53: delete “,” after intra-. Corrected (Line 54)

-- Line 59: Bioinformatic. Corrected (Line 60)

-- Line 81: CdgB DGC and PDE activity under same conditions or different? Please mention. Corrected (Line 82)

-- Line 85: What is PGPR? First time used abbreviation should be elaborated. Corrected (Line 87)

-- Line 113: Gentamicin. Corrected (Line 114)

-- Line 245-46: How did you measure the color intensity? Please mention. Corrected, we added the following text: The relative fluorescence intensity (RFI) was calculated as the ratio between the TurboRFP and AmCyan fluorescence intensities and is directly proportional to c-di-GMP levels, as analyzed using ImageJ software. The RFI values represent the standard deviations of three biological replicates, and significant differences are indicated at * P < 0.05 according to Student´s t-test by SigmaPlot as previously described (Lines 257-261)

-- Line 311: proposed to be… Corrected (Line 320)

-- Line 449: riboswitches? Or riboswitch? Please specify one or multiple. Corrected (Line 242)

-- Line 508: *Bacillus subtilis*, although… Corrected (Line 594)

**Major comments**

**Request 1**

-- Line 144: For overexpression of *cdgB* was cloned along with its native promoter? Or the ORF of the gene was cloned in frame under the p*lac*? If the native promoter was included, then there is a possibility of additional regulation at the *cdgB* promoter. The phenotypes observed in *A. baldaniorum* might include additional effects of *cdgB* transcriptional regulation. The experiments with overexpression of *cdgB* will benefit from constitutive expression under *lac* promoter.

**Response 1**

*cdgB* gene overexpression is under the control of the lac promoter only. We corrected the mistake. The full-length ORF of *cdgB* gene (Line 145).

**Request 2**

**Additional suggestion:** to support the expression of all the native and mutated variants of CdgB, a western blot of tagged protein would be strong evidence for full expression of the protein variants. This would confirm that the phenotypes observed was not because of any artifacts.

**Response 2**

We agree that without the requested evidence, it is not possible to discard potential stability effects of introducing point mutations into *cdgB*. Since we do not have specific antibodies to detect these proteins it would not be possible to provide these data in time for the revision deadline. However, multiple reports have used the same type of point mutations in DGC-PDE proteins with no effect on stability. We did analyze the stability of the fusions CdgB-eGFP full-length and CdgB_ΔMHYT_-eGFP by western blot using an eGFP antibody (S4 figure) (Lines 205, 416-417) and did not observed differences between them. In the revised version of the manuscript we show that the fusion protein expressed from pMP-*cdgB*::*egfp* is functional in biofilm and Congo Red assays (figures 4 and 5).

**Request 3**

-- Fig 4, 5 and 7: Biofilm, EPS production and swimming assay- the T test and the annotations on the graph for statistical significance is confusing and doesn’t compare all the samples tested. A One-way ANOVA followed by a multiple comparison test (Like Tukey’s test) would actually compare all the sample sets and give the readers a clear idea of the statistically significant differences among each of the samples. Also, was T-test performed between each of the samples or just between each control and their corresponding tests samples? Need a mention. The asterisks and the labels above the bars in the graphs are not clear which samples are being compared. This can be explained briefly in figure legends.

**Response 3**

In response to the reviewer comments, we remade the plots from figures 4, 5 and 7. We deleted the marks on plots and compared each control with their corresponding tests samples.

**Request 4**

-- The c-di-GMP differences in various strains and overexpression of *cdgB* was measured mostly by qualitative methods. A quantitative measurement of cellular c-di-GMP would be helpful to understand the dominance of either DGC or PDE activity of CdgB under different conditions.

**Response 4**

In response to the reviewer comments, in the revised version of the manuscript we provide quantitative values for the results obtained using the c-di-GMP reporter (See figure 8). To quantify the fluorescence of cells from fluorescent images, we used a previously reported method described by (Cruz-Pérez et al 2021 and Shihan M et al 2021). The excitation and emission settings for the detection of Amcyan and TurboRFP were 457/520 nm and 553/574 nm, respectively. The relative fluorescence intensity (RFI) was calculated by dividing the arbitrary fluorescent intensity units of TurboRFP by those of Amcyan as previously described (Cruz-Pérez et al 2021; Shihan MH et al 2021.) Experiments were repeated at least three times.

References

Cruz-Pérez JF, Lara-Oueilhe R, Marcos-Jiménez C, Cuatlayotl-Olarte R, Xiqui-Vázquez ML, Reyes-Carmona SR, Baca BE, Ramírez-Mata A. Expression and function of the cdgD gene, encoding a CHASE-PAS-DGC-EAL domain protein, in Azospirillum brasilense. Sci Rep. 2021 Jan 12;11(1):520. doi: 10.1038/s41598-020-80125-3. PMID: 33436847; PMCID: PMC7804937.

Shihan MH, Novo SG, Le Marchand SJ, Wang Y, Duncan MK. A simple method for quantitating confocal fluorescent images. Biochem Biophys Rep. 2021 Feb 1;25:100916. doi: 10.1016/j.bbrep.2021.100916. PMID: 33553685; PMCID: PMC7856428.

**Request 5**

-- A quantification of cellular c-di-GMP level in *A. baldanorium* strains is required. The differences of phenotypes (like biofilm, swimming, etc.) observed in the strains because of deletion or overexpression of *cdgB* is caused by variation of c-di-GMP level, needs to be established.

**Response 5**

We agree it is important to optimize a methodology to quantify c-di-GMP levels in *A. baldinorium*. We could not use the same fluorescent c-di-GMP biosensor that allowed us to detect c-di-GMP levels in *E. coli* because the plasmid used for overexpression of *cdgB* in *A. baldinorium* is of the same incompatibility group as the plasmid that harbors the c-di-GMP biosensor. We are beginning the quantitative standardization of c-di-GMP level by HPLC coupled to mass spectrometry. In a future work we are planning to measure c-di-GMP levels in *A. baldanorium* cells grown under different conditions, such as presence and absence of NO, O_2_, CO_2_ and other type of signals.

**Request 6**

-- The DGC activity of CdgB increased by overexpression in *E. coli* but the reverse in *A. baldanorium.* Is it host specific? Requires an elaboration in the discussion section. A quantitative measurement of cellular c-di-GMP can be done with overexpression of *cdgB* in both *A. baldanorium* and *E. coli* under the same or similar condition and a comparison can be shown to determine the concentration-dependent catalytic activity of CdgB. Then conclusion can be made whether the DGC activity of CdgB in *E. coli* is host-specific or conditional.

**Response 6**

We consider the observation that CdgB acts mainly as a DGC in *E. coli* as evidence of its capability of dual activity. The factors that affect the activity switch will be explored in future work. In *A. baldanorium* it appears that the abundance of CdgB could affect the DGC-PDE switch. The absence of CdgB results in a decrease in biofilm formation, a phenotype that is positively influenced by c-di-GMP. This would suggest that at baseline levels CdgB may act as a DGC. When *cdgB* is overexpressed in a genetic background that has a native copy of *cdgB*, biofilm formation is also inhibited. This would suggest that in these conditions CdgB has switched from DGC to PDE activity. Interestingly, when a variant with a putative dead PDE domain was overproduced biofilm formation was significantly induced. This strongly suggests that CdgB can switch between the two activities in *A. baldanorium*. We speculate that the different behavior of CdgB in *E. coli* could be due to differences in nitrogen metabolism. Recently Park et al 2020 demonstrated that a nitrite transporter stimulated biofilm formation by controlled NO production via appropriate nitrite reductase suppression and subsequent diguanylate cyclase activation, and c-di-GMP production in *E. coli* and *P. aeruginosa*. Additionally, *E. coli* possesses a transcription factor named NorR with a non-heme iron center to sense NO (D'Autréaux B, et al 2005), indication of different regulation mechanisms. More work needs to be done to elucidate what could be favoring the DGC activity of CdgB in *E. coli* (Lines 583-591)

Reference

Park JS, Choi HY, Kim WG. The Nitrite Transporter Facilitates Biofilm Formation via Suppression of Nitrite Reductase and Is a New Antibiofilm Target in Pseudomonas aeruginosa. mBio. 2020 Jul 7;11(4):e00878-20. doi: 10.1128/mBio.00878-20. PMID: 32636243; PMCID: PMC7343986.

D'Autréaux B, Tucker NP, Dixon R, Spiro S. A non-haem iron centre in the transcription factor NorR senses nitric oxide. Nature. 2005 Sep 29;437(7059):769-72. doi: 10.1038/nature03953. PMID: 16193057.

**Request 7**

-- Deletion of *cdgB* resulted in a different phenotype compared to overexpression in *A. baldanorium*. This suggests that high cellular concentration of CdgB increases its PDE activity in *A. baldanorium*. This means that increased transcription and subsequently translation of *cdgB* is responsible for reduced c-di-GMP in the bacterium in response to the environment. An experiment showing transcription of *cdgB* and also its translation under different conditions such as presence and absence of NO, O_2_ and CO_2_ followed by measurement of intracellular c-di-GMP levels in *A. baldanorium*. It can be beneficial and complement the data shown in the manuscript. This experiment can be used to determine if the PDE and DGC activity of CdgB is dependent on its intracellular concentration or environmental signals, since the signals perceived by this protein is to be determined in future.

**Response 7**

The reviewer’s suggestion is very interesting. Our goal for this manuscript is to be the anchor point for future analysis. The mater of the regulation of *cdgB* is one of our current interests. It is unclear if *cdgB* has its own promoter upstream or if it is co-transcribed with its neighbor genes, a putative *lysR* gene upstream and a gene of unknown function downstream (see S1 Figure) (Lines 892-894). Together with an in-depth characterization of the regulatory region of *cdgB,* as the reviewer suggested, we are looking to analyze how its expression is affected by signals that could be detected by the MHYT domains, such as NO, O_2_ and CO_2_.

**Request 8**

Explain clearly on what is the difference between CdgB and previously reported GGDEF-EAL proteins in the discussion.

The first characterized hybrid protein DGC-PDE with an MHYT domain was YkoW from *Bacillus subtilis* although the involvement of this domain in signal perception and enzymatic function could not be ascertained. Nevertheless, it was proposed that the MHYT domain could potentially sense O_2_, CO, or NO through the coordination of one or two copper atoms [46]. Other proteins characterized to date with an MHYT domain are NbdA and MucR from *P. aeruginosa* both implicated in NO-mediated biofilm dispersion. The deletion of *nbdA* and mucR impaired NO-induced biofilm dispersion, only NbdA appeared to be specific to the NO-induced dispersion response. Although MucR was shown to play a role in biofilm dispersion in response to NO and glutamate, exposure to NO in the presence of MucR but the absence of NbdA did not result in increased PDE activity. MucR is one of the few proteins harboring both EAL and GGDEF domains that possess both DGC and PDE activity [47,67]. Furthermore, MucR is proposed to interact with the alginate biosynthesis protein Alg44, which contains a c-di-GMP binding PilZ domain essential for alginate biosynthesis [5,67]. Recently, other MucR homologous protein was described in *Azotobacter vinelandii* implicated in the alginate biosynthesis too [73]. (Lines 597-608)

**Request 9**

Predict the function of CdgB in the mutualistic relationships between Azospirillum baldaniorum and plants.

NO has been shown to promote root growth promotion[75] and biofilm formation in *A. baldaniorum* Sp245, the absence of a periplasmic nitrate reductase (Nap) significantly affects biofilm formation through a mechanism yet to be explored [40]. Since the MHYT domain has been proposed to sense NO it is tempting to speculate that CdgB could participate in a singling module that controls biofilm formation in response to nitric oxide. We speculate that NO may be sensed by the MHYT domain of CdgB. Reception of this signal may inﬂuence CdgB activity by modulating DGC activity and possibly PDE domain activity. Nevertheless, the molecular details of this mechanism remain largely unknown, and need more analyses. (Lines 617-625)
